# Gene Regulatory Network that Shaped the Evolution of Larval Apical Organ in Cnidaria

**DOI:** 10.1093/molbev/msad285

**Published:** 2023-12-28

**Authors:** Eleanor Gilbert, Jamie Craggs, Vengamanaidu Modepalli

**Affiliations:** Marine Biological Association of the UK, The Laboratory, Citadel Hill, Plymouth PL1 2PB, UK; School of Biological and Marine Sciences, University of Plymouth, Plymouth PL4 8AA, UK; Horniman Museum and Gardens, London SE23 3PQ, UK; Marine Biological Association of the UK, The Laboratory, Citadel Hill, Plymouth PL1 2PB, UK

**Keywords:** apical organ, evolution, cilia, cnidaria, *Aurelia*, *Nematostella*, *Acropora*

## Abstract

Among non-bilaterian animals, a larval apical sensory organ with integrated neurons is only found in cnidarians. Within cnidarians, an apical organ with a ciliary tuft is mainly found in Actiniaria. Whether this apical tuft has evolved independently in Actiniaria or alternatively originated in the common ancestor of Cnidaria and Bilateria and was lost in specific groups is uncertain. To test this hypothesis, we generated transcriptomes of the apical domain during the planula stage of four species representing three key groups of cnidarians: *Aurelia aurita* (Scyphozoa), *Nematostella vectensis* (Actiniaria), and *Acropora millepora* and *Acropora tenuis* (Scleractinia). We showed that the canonical genes implicated in patterning the apical domain of *N. vectensis* are largely absent in *A. aurita*. In contrast, the apical domain of the scleractinian planula shares gene expression pattern with *N. vectensis*. By comparing the larval single-cell transcriptomes, we revealed the apical organ cell type of Scleractinia and confirmed its homology to Actiniaria. However, *Fgfa2*, a vital regulator of the regionalization of the *N. vectensis* apical organ, is absent in the scleractinian genome. Likewise, we found that *FoxJ1* and 245 genes associated with cilia are exclusively expressed in the *N. vectensis* apical domain, which is in line with the presence of ciliary apical tuft in Actiniaria and its absence in Scleractinia and Scyphozoa. Our findings suggest that the common ancestor of cnidarians lacked a ciliary apical tuft, and it could have evolved independently in the Actiniaria.

## Introduction

**Fig. 1.**
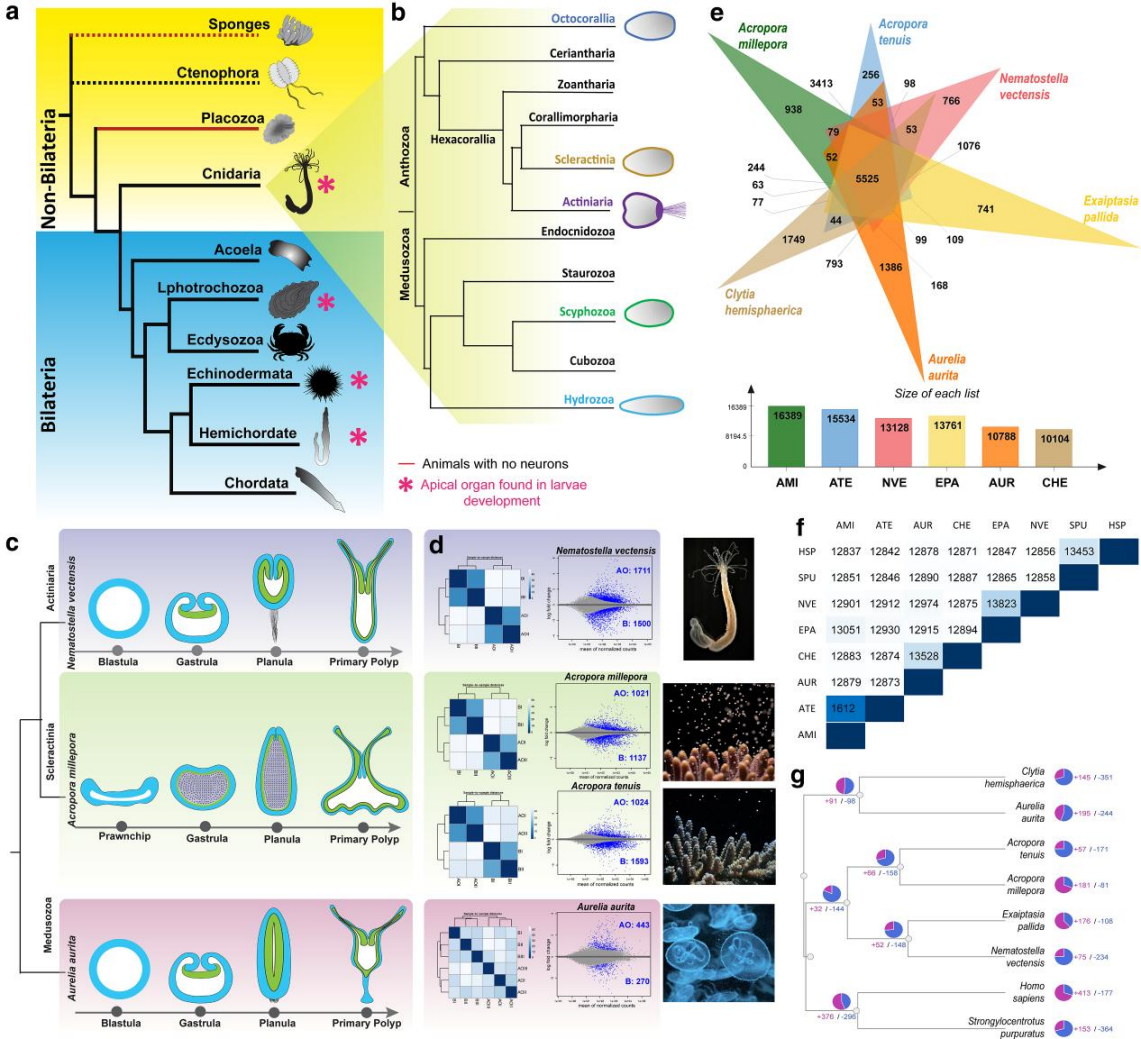
Evolutionary origin of the apical organ and tissue-specific transcriptome of the cnidarian apical organ. a) A brief overview of evolutionary relationships in the animal kingdom. Within non-bilaterians, a true larval apical organ with integrated neurons is only found in cnidarians. b) An overview of evolutionary relationships in the phylum Cnidaria; an evident long ciliary apical tuft is commonly found in Actiniaria. c) Schematic drawing of embryonic development in different cnidarian taxa. The fertilized eggs progress through gastrulation and to planula. Planula larvae, after metamorphosis, transform into a feeding primary polyp. d) MD and volcano plots represent the logFC ratio of differential expression between apical and body tissues from respective species. The significantly differentially expressed genes are highlighted in blue. MD plots display a global overview of all data sets. AO: apical organ; B: planula body. e) A Venn diagram of orthologous genes shared between cnidarian species. The size of clusters in each species, including orthologs and in-paralogs. f) Summary of proteins overlapped across each species. g) The phylogenetic relationship of a selected list of cnidarians and bilaterian species was inferred based on ortholog gene groups and included the number of expanded and contracted ortholog groups indicated by CAFE analysis. Species abbreviations: AAU: *Aurelia aurita*, AMI: *Acropora millepora*, ATE: *Acropora tenuis*, CHE: *Clytia hemisphaerica*, EPA: *Exaiptasia pallida*, NVE: *Nematostella vectensis*, HSP: *Homo sapiens*, SPU: *Strongylocentrotus purpuratus*.

The majority of marine benthic invertebrates progress through a planktonic life phase during early development, a ciliated larva with an apical organ (Marinković et al. 2020). The apical organ is a larval neurosecretory structure identified in diverse groups of marine invertebrates and located at the frontal region of the larvae. Behavioral studies have demonstrated that ciliated larvae use the apical organ to process environmental cues and modulate their swimming behavior (Schmich et al. 1998; Iwao et al. 2002; Sinigaglia et al. 2015; Bezares-Calderón et al. 2023). The apical domain of the larvae is often equipped with an “apical tuft” of long cilia that protrude from apical cells (Chia and Koss 1979; Page 2002; Gruhl 2009; Conzelmann et al. 2011; Goldberg et al. 2011; Garner et al. 2015; Verasztó et al. 2017; Marinković et al. 2020). Among non-bilaterians (Cnidaria, Placozoa, Porifera, and Ctenophora), a larval sensory organ with integrated neurons is only found in cnidarians (Fig. 1a) (Marlow et al. 2009; Kelava et al. 2015; Layden et al. 2016).

A more profound question is the evolutionary origin of apical organs, whether the apical organs of ciliated larvae across different phyla are homologous or evolved convergently. The morphology of the apical organ in cnidarian larvae is comparable to those of bilaterian larvae (Marlow et al. 2014; Nielsen 2015; Sinigaglia et al. 2015), indicating that the common ancestor of these two groups may have progressed through a free-swimming larval stage with a true larval apical organ and associated neurons (Nielsen 2005, 2013). A highly conserved set of genes patterning the apical/anterior ectoderm in Bilateria and in the apical/aboral ectoderm in cnidarian *Nematostella vectensis* shows that these regions are very likely homologous (Santagata et al. 2012; Range et al. 2013; Sinigaglia et al. 2013; Marlow et al. 2014; Kitzmann et al. 2017; Lebedeva et al. 2021). Genes such as *Six3/6*, *FoxJ*, *FoxQ2*, *Irx*, *Rx*, *Hox*, *Nkx3*, and *Frizzled* are shared between cnidarian *N. vectensis* and bilaterian species (Santagata et al. 2012; Range et al. 2013; Sinigaglia et al. 2013; Marlow et al. 2014; Kitzmann et al. 2017; Lebedeva et al. 2021). This, in turn, makes it essential to consider the possible “deep homology” of the apical organs across different phyla. Given the remarkable fraction of transcriptional factors (TFs) of the apical gene regulatory network (GRN) that also contribute to the development of other nervous systems of various forms and functions (Holland et al. 2013; Arendt et al. 2016; Layden 2019; Faltine-Gonzalez et al. 2023; Gattoni et al. 2023), it is conceivable that regulatory modules that are commonly expressed in animals with more complex nervous systems are also deployed in cnidarian larvae. Strikingly, many TFs associated with nervous system development have demonstrated a conserved spatial distribution across the anterior–posterior axis of the nervous system among cnidarian *N. vectensis* and bilaterian species (Arendt et al. 2016; Faltine-Gonzalez et al. 2023). Whether these homologous TFs can also be recognized across different cnidarian taxa is unclear.

Cnidarians are divided into two major groups: Anthozoa (sea anemones, corals, and sea pens) and Medusozoa (jellyfish, sea wasps, and *Hydra*) (Fig. 1b). Even though cnidarians are monophyletic, the magnitude of genetic differences between the anthozoans and medusozoans is equivalent to that between Anthozoa and Deuterostomia (Putnam et al. 2007; Khalturin et al. 2019a). Molecular dating estimated the separation of the major cnidarian clades more than 500 MYA, and each group has undergone a long period of independent evolution (Khalturin et al. 2019a). Though a large proportion of early embryonic development is shared across these groups, the actiniarian planula, like *N. vectensis*, is morphologically unique as it possesses a long ciliated apical tuft (Fig. 1b). The apical domain displays several apical cells bearing a ciliated tuft as well as RPamide and PRGamide peptidergic cells (Zang and Nakanishi 2020; Gilbert et al. 2022). In Medusozoa and Scleractinia, the apical domain is also enriched with neuropeptide-expressing cells (Leitz and Lay 1995; Gajewski et al. 1996; Iwao et al. 2002; Katsukura et al. 2004; Piraino et al. 2011). However, unlike Actiniaria, the Scleractinia and Medusozoa commonly lack a long ciliated tuft. Exceptionally, some non-reef building coral species such as *Astrangia poculata* in the Rhizangiidae family of the Scleractinia have been documented with a ciliary apical tuft (Warner et al. 2023). The widespread occurrence of apical organ in multiple phyla of marine invertebrates (Fig. 1a and b) and clade-specific absence has prompted radically different views on the origin of the apical organ. Some consider them an ancient feature of the eumetazoan ciliated larvae (Jagersten 1972; Davidson et al. 1995; Peterson et al. 1997; Nielsen 2012). Alternatively, others argue that they could have evolved multiple times independently (Wolpert 1999; Sly et al. 2003; Raff 2008; Martín-Zamora et al. 2023). Within cnidarians, the inconsistency of apical organ structures and the lack of apical tuft in the frontal region of scleractinian and jellyfish larvae has raised doubt over the common origin of the apical organ and the advantages of long ciliated tuft in specific groups. (Figure 1b). Whether an apical organ with a ciliary tuft is ancestral or has evolved convergently remains unresolved, and the regulatory genes that facilitated the evolution of the apical tuft in the Actiniaria are yet to be addressed.

Here, we performed tissue-specific transcriptomics to compare the molecular basis of the apical domain in three groups of cnidarians, including Actiniaria (sea anemones), Scleractinia (stony corals), and Scyphozoa (true jellyfishes). This technique has proven robust in identifying the apical enriched genes in our previous study in *N. vectensis* (Gilbert et al. 2022). We examined the spatial distribution of transcription factors and other key signaling components previously studied in the frontal/apical region of the larvae (Matus et al. 2007a; Mazza et al. 2007; Rentzsch et al. 2008; Nakanishi et al. 2012; Marlow et al. 2013; Richards and Rentzsch 2014; Watanabe et al. 2014; Sinigaglia et al. 2015; Leclère et al. 2016; Gilbert et al. 2022). We revealed the evolutionary relationship of apical organ between Cnidaria groups.

## Results

### Tissue-specific Transcriptome of Cnidarian Planula Reveals the Molecular Topology Tightly Associated With the Apical Domain

To reveal the gene expression profile of the larval apical domain among cnidarian groups, we systematically carried out microdissections on cnidarian larvae collected at the planula stage from Actiniaria *Nematostella vectensis*, Scyphozoa *Aurelia aurita* and two Scleractinia species *Acropora millepora* and *Acropora tenuis* (Fig. 1c). We carefully separated the apical tissue from the rest of the larval body and acquired transcriptomic data from both the apical and the rest of the body tissues separately to perform differential gene expression (DGE) analysis. By using DGE analysis, we identified significantly differentially expressed genes (DEGs) between the apical and body tissue of each species (Fig. 1d). The global gene expression patterns among the apical and body tissues from replicates were compared using principal component analysis and correlation analysis (Fig. 1d); the plots displayed a strong correlation among the replicates. Notably, *A. aurita* presented a relatively low number of significantly DEGs: 713 [*p*_adj_ (FDR) < 0.05]. Of all four species, the Actiniaria *N. vectensis* presented the highest number of significantly DEGs (3225) between apical and body tissues. The number of significantly DEGs for each species is presented in Fig. 1d, supplementary file S1, Supplementary Material online.

### Orthology Analyses to Compare the Planula Anteroposterior Molecular Composition Between Actiniaria, Scyphozoa and Scleractinia Larvae

**Fig. 2.**
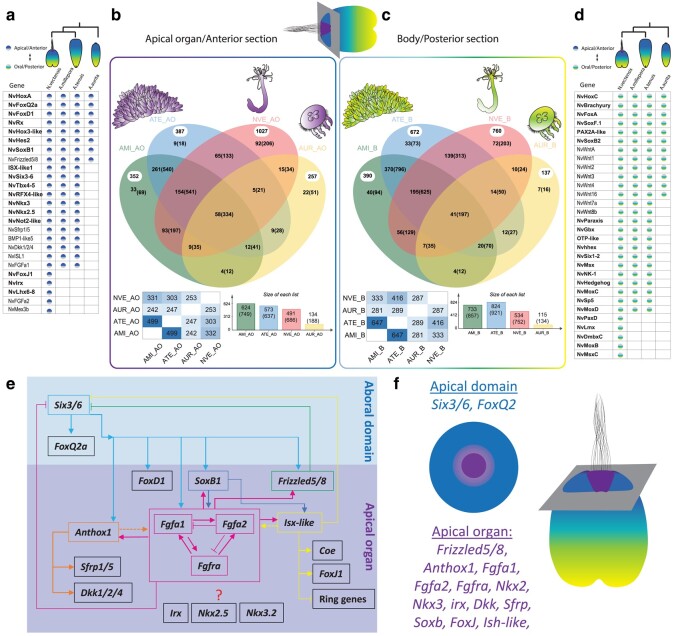
Comparison of tissue-specific genes with shared expression between cnidarian planulae. a, d) The table detailing each TF (bold) and other critical developmental genes enriched in apical domain (a) and body section of planula (d) from *N. vectensis*, *A. millepora*, *A. tenuis* and *A. aurita*. b, c) Venn diagrams presented the distribution of shared and unique orthogroups between *N. vectensis*, *A. millepora*, *A. tenuis* and *A. aurita* in apical domain (b) and the body section of planula (c). The number of proteins in ortholog groups/clusters are indicated in brackets next to the number of ortholog groups/clusters. The number in white circles indicates the number of singletons. At the bottom presented the summary of proteins overlapped across each species and the size of clusters in each species, including orthologs and in-paralogs. e) Illustrating *N. vectensis* apical domain and oral/posterior domain GRN (Marlow et al. 2013; Sinigaglia et al. 2013, 2015; Watanabe et al. 2014; Layden et al. 2016; DuBuc et al. 2018; Technau 2020; Lebedeva et al. 2021; Gilbert et al. 2022; Schwaiger et al. 2022; Sabin et al. 2023). f) Segregation of expression domains in *N. vectensis* planula apical domain divided into ring and spot territories (Sinigaglia et al. 2013, 2015; DuBuc et al. 2018; Gilbert et al. 2022; Sabin et al. 2023). Species abbreviations: AAU: *Aurelia aurita*, AMI: *Acropora millepora*, ATE: *Acropora tenuis*, NVE: *Nematostella vectensis*.

To enable comparison of planula anteroposterior patterning among different groups of Cnidaria, we reveal the molecular composition of anteroposterior domains in *N. vectensis*, *A. millepora*, *A. tenuis*, and *A. aurita* using the spatial gene expression data. Identifying orthologous clusters is critical for comparative genomic studies, as it facilitates the comparison of evolutionary relationships between genes across different species. Therefore, as a first step in our comparison across cnidarian groups, we implemented orthology analyses to group the cnidarian proteomes to identify both species-specific and homologous genes (Fig. 1e–g). Along with *N. vectensis*, *A. millepora*, *A. tenuis*, and *A. aurita*, we additionally included cnidarians *Clytia hemisphaerica* and *Exaiptasia pallida* as well as bilaterians *Strongylocentrotus purpuratus* and *Homo sapiens.* The output data from the orthology analyses are provided in supplementary file S2, Supplementary Material online. Next, we sorted the genes enriched in apical and body tissues from DEGs data into two groups. Using OrthoVenn3, we identified orthologous groups shared across four species in apical and body tissues separately (Fig. 2, supplementary file S3, Supplementary Material online). Within apical tissue enriched gene sets, 58 orthogroups with 314 proteins were expressed in all four species, 152 orthogroups (522 proteins) were shared across anthozoan species, and 261 orthogroups (540 proteins) were shared exclusively between the scleractinian *A. millepora* and *A. tenuis*. 92 orthogroups (206 proteins) were expressed only in the *N. vectensis* apical domain (Fig. 2b). *N. vectensis* also expressed 1,027 singletons exclusively in the apical domain. As detailed in Fig. 2c, we also defined co-expression dynamics of genes in the posterior domain or body tissue across *N. vectensis*, *A. millepora*, *A. tenuis* and *A. aurita* through orthology analyses (Fig. 2c).

### Despite the Lack of Apical Tuft, Scleractinian Planulae Share Conserved Anteroposterior Patterning Genes With Actiniaria

Scleractinian corals belong to the Hexacorallia, a lineage within the class Anthozoa and a sister group to the Actiniaria (Fig. 1b). However, the frontal region of the scleractinian planula differs from the actiniarian planula mainly due to the absence of the ciliary apical tuft. The extent to which the Scleractinia apical domain shares molecular topography with its sister group Actiniaria is yet to be addressed. The frontal region of cnidarian embryos is set by an apical domain GRN directing the formation of apical organ. The well-defined developmental GRN in *N. vectensis* provides a powerful framework for investigating the evolution of embryonic patterning mechanisms (Rentzsch et al. 2008; Marlow et al. 2009; Neugebauer et al. 2009; Richards and Rentzsch 2014; Watanabe et al. 2014; Leclère et al. 2016; Technau 2020; Lebedeva et al. 2021; Gilbert et al. 2022; Sabin et al. 2023).

If the apical domain is evolutionarily related across cnidarians, an overlap of GRNs would be expected. Using spatial transcriptome data and comparative genomics, we first sought to define the co-expression dynamics of genes involved in the patterning of anteroposterior territories along the planula apical and body tissues (supplementary File S1, Supplementary Material online). In *N. vectensis*, the apical domain GRN is composed of *NvIsx-like*, *NvFgfa1*, *NvFgfa2*, *NvSfrp*, *NvFrizzled-like*, *NvDickkopf-like*, *NvNkx3*, *NvNkx2.5*, *NvTbx4-5*, *NvHoxA* (*Anthox1*), *NvFoxQ2a, NvIrx*, *NvFoxD1*, *NvRX, NvHox3-like*, *NvHes2*, *NvSoxB1*, *NvSix3-6*, *NvRFX4-like*, *NvFoxJ1*, *NvTauD*, *PoxA-like* and *Bmp1-like5* genes. Chiefly, *NvHoxA* (*Anthox1*), *NvFgfa1*, *NvFgfa2*, *NvSix3-6*, and *NvIsx-like* have been functionally studied, and the knockdown (KD) of each of these genes independently has resulted in the ether loss of apical tuft cells or defects in apical organ development (Rentzsch et al. 2008; DuBuc et al. 2018; Gilbert et al. 2022). Using spatial transcriptome data, we compared the shared expression of these orthologs in the Scyphozoa and Scleractinia planula apical domain. As illustrated in Fig. 2a, genes that pattern apical domain of *N. vectensis* are largely expressed in similar patterns in Scleractinia *A. millepora* and *A. tenuis* planula this includes *Isx-like*, *Fgfa1*, *Fgfa2*, *Sfrp*, *Frizzled-like*, *Dickkopf-like*, *Nkx3*, *Nkx2.5, Tbx4-5*, *HoxA* (*Anthox1*), *FoxQ2a, FoxD1, Rx, Six3-6, Rfx4-like, FoxJ1, Hes2, SoxB1, PoxA-like* and *Bmp1-like5* genes. Despite the lack of apical tuft, Scleractinia planulae share a similar set of genes in their apical domain, suggesting that these TFs already operated as a part of a GRN that was assembled before the separation of Actiniaria and Scleractinia.

In parallel, we also compared the genes associated with posterior domain by using previously characterized genes from *N. vectensis* studies (Fig. 2). We compared the posterior region with the genes including *NvHoxC, NvBarH*, *NvFoxA*, *NvSoxF*, *NvGbx*, *NvMsx*, *NvHedgehod*, *NvMoxA, NvMoxB, NvMoxC, NvMoxD*, *NvPaxD*, *NvLMX*, and members of the *Wnt* (*NvWntA*, *NvWnt1*, *NvWnt3*, *NvWnt4*, *NvWnt16*, *NvWnt2,* and *NvWnt8*). Note that with some of the TFs, we could only find significant differential expression in one of the *Acropora* species; therefore, we excluded them from the list (supplementary file S1, Supplementary Material online). As illustrated in Fig. 2d, genes that pattern the posterior territory of *N. vectensis* are largely expressed in similar patterns in Scleractinia *A. millepora* and *A. tenuis* planula, including *HoxC* (*Anthox6*), *BarH*, *FoxA*, *SoxF*, *Gbx*, *Msx*, *Hedgehog, MoxC, MoxD,* and members of the *Wnt* family (*WntA*, *Wnt1*, *Wnt3*, *Wnt4*, *Wnt16*, *Wnt2,* and *Wnt8*). This reveals that Scleractinia and Actiniaria share a large portion of GRN in both the anterior and posterior domains.

**Fig. 3.**
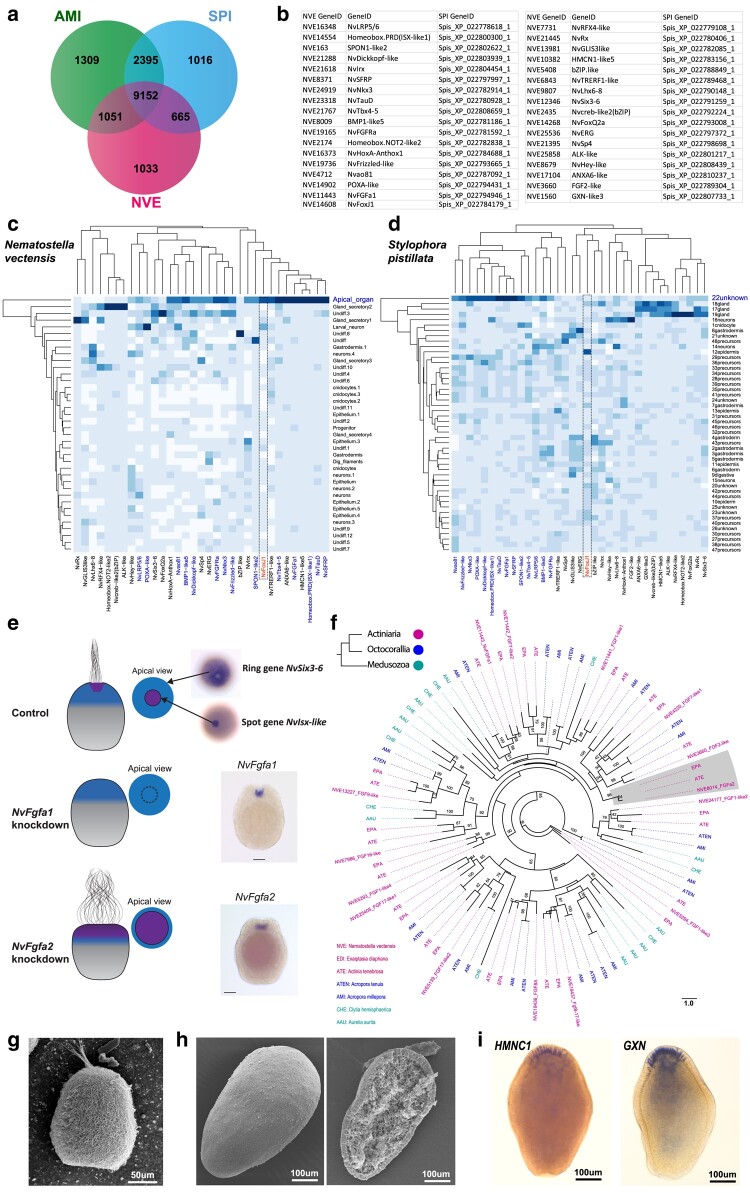
Defining apical organ in scleractinia planula. a) Venn diagrams presenting the distribution of shared and unique orthogroups between *N. vectensis*, *A. millepora* and *S. pistillata*. b) A table detailing the homologs of *N. vectensis* apical domain genes identified in *S. pistillata* and their respective gene IDs. c, d) Heatmaps displaying the gene expression of selected apical domain marker genes across larval cell types classified through single-cell transcriptomes of *N. vectensis* (c) and *S. pistillata* (d). e) Schematic drawings illustrating the morphological phenotype after *NvFgfa1* and *NvFgfa2* KD. *NvFgfa1* KD produces larvae lacking an apical organ and ciliary tuft, while *NvFgfa2* KD leads to larvae with an expanded apical organ and ciliary tuft. f) A phylogenetic relationship of cnidarian FGF proteins. Values on nodes represent Bootstrap values (100 replicates). Bootstrap support values above 50% are indicated above branches. g) SEM of *N. vectensis* planula viewed laterally. h) SEM of *A. millepora* planula viewed laterally, images on the right; the planula was cracked open to visualize the internal structures. i) The expression of the top two genes *HMNC1* (XP_044178031.1) and *GXN* (XP_029194558.2), enriched in the *A. millepora* apical domain, was determined by ISH. Species abbreviations: AMI: *Acropora millepora*, NVE: *Nematostella vectensis*, SPI: *Stylophora pistillata*.

The cellular identity of apical organ has never been detailed in scleractinian larvae. A previously published study of larval single-cell transcriptome in the scleractinian *Stylophora pistillata* has not classified apical organ cell types (Levy et al. 2021). From the tissue-specific transcriptome and orthology analyses, we revealed the shared apical domain genes in scleractinian planula. Using characterized *N. vectensis* apical organ marker genes and single-cell transcriptome data (Sebe-Pedros et al. 2018), in combination with orthology analyses, we investigated the Scleractinia planula single-cell data (Levy et al. 2021) to predict apical cell types. We first identified the orthologs shared across *N. vectensis*, *A. millepora,* and *S. pistillata* (Fig. 3a, supplementary file S4, Supplementary Material online). Next, based on previous studies in *N. vectensis*, we selected apical organ genes including *NvIsx-like*, *NvFgfa1*, *NvFgfa2*, *NvFgfra*, *NvSfrp*, *NvTauD*, *NvFrizzled-like*, *NvDickkopf-like*, *PoxA-like*, *Bmp1-like5*, *NvNkx3*, *NvNkx2.5, NvLhx6-8*, *NvTbx4-5*, *NvHoxA* (*Anthox1*), *NvFoxQ2a,* and *NvIrx* (Fig. 3b, supplementary fig. S2, Supplementary Material online). We also included genes expressed in the apical domain enriched cell types such as gland cells, larval specific neurons and undifferentiated cell type 3: *NvRx*, *Hmcn1-like5*, *NvSix3-6*, *Klkb1-like3*, *NvRfx4-like*, *Anxa6-like*, *Nvcreb-like2*, *Dbp-like2* and *NvTrerf1-like* (Fig. 3c). Next, we pulled out the expression patterns of the list of genes associated with the *N. vectensis* apical organ. From the comparison, we identified a set of apical organ genes that are specifically expressed in an undefined cell type 22 (Fig. 3d). This cell type displayed shared expression of some of well characterized *N. vectensis* apical organ genes, including *Frizzled-like*, *Sfrp*, *Lrp5/6*, *Isx-like*, *Dickkopf-like*, *Nkx3*, *TauD*, *Fgfa1*, *Fgfra*, *Bmp1-like5*, *Tbx4-5*, *Spon1-like*, and *PoxA-like* (Fig. 3d). This analysis reveals the apical cell types (cluster 22) in *S. pistillata* and provides additional evidence on shared apical organ cell types between actiniarian and scleractinian species.

Of all the apical domain genes, we noted that *Fgfa2*, a critical regulator of *N. vectensis* apical organ, is primarily absent in the *A. millepora* and *A. tenuis* differential expression data. Furthermore, from the FGF phylogenetic analysis, we identified that *Fgfa2* is absent in the Scleractinian genomes (Fig. 3f, supplementary file S5, Supplementary Material online). Earlier studies in *N. vectensis* have revealed that, the *N. vectensis* genome encodes 15 homologous transcripts of Fibroblast growth factors (Fgfs) and two Fibroblast growth factor receptors (Fgfrs). Furthermore, it has been demonstrated that two paralogous FGF genes (*NvFgfa1* and *NvFgfa2*) and one FGF receptor gene (*NvFgfra*) are expressed in the apical domain of *N. vectensis* with a spot expression pattern (Rentzsch et al. 2008; Sinigaglia et al. 2013). Phylogenetically, *NvFgfa1* and *NvFgfa2* belong to an eight-membered paralogous group which cannot be assigned with certainty to a particular subfamily. Within this paralogous group, *NvFgfa1* and *NvFgfa2* are distantly related (Matus et al. 2007b; Rentzsch et al. 2008). Functional studies demonstrate that the development of the apical ciliary organ in *N. vectensis* is under the control of *NvFgfa1*, *NvFgfa2*, and *NvFgfra* (Rentzsch et al. 2008; Sinigaglia et al. 2013) (Fig. 3e). As illustrated in Fig. 2f, the aboral domain surrounding and encompassing the apical tuft structure reveals two major concentric domains. The first external circle in the apical domain expressing *NvSix3/6*, *NvFoxQ2a*, and *NvFoxD1* are devoid from apical tuft/apical organ territory (Fig. 2e and f, blue color). While *NvFgfa1*, *NvFgfa2*, *NvFgfra, NvIrx*, *NvFoxJ* and *NvIsx-like* are restricted mainly to the spot region of apical domain, this also includes other spot genes *Dhh*, *NvSfrp1/5*, *NvNkx2.1*, *NvNkx3.5*, *NvSoxB* and *Anthox1* (Sinigaglia et al. 2013) (Fig. 2e and f, purple color). KD experiments show that the signaling of *NvFgfa1* is required for the specification of the apical organ's ciliary tuft. In contrast, *NvFgfa2* is necessary to limit the size of ciliary tuft cells to spot regions by the antagonistic interplay of *NvFgfa1* and *NvFgfa2* signaling (Fig. 3e) (Rentzsch et al. 2008; Sinigaglia et al. 2013). The morphology of the scleractinian planula differ from the anthozoan planula at the frontal region and somewhat resembles a *NvFgfa2* KD planula (Fig. 3e and h–i). Similarly, based on the expression of the top two genes HMNC1 and GXN, enriched in the *A. millepora* apical domain, the cells in this region displayed a wide distribution. It seems *NvFgfa2* is an actiniarian innovation to restrain the apical cells to spot area of the apical domain and this extensive patterning of the apical domain is unique to anthozoan clade. However, a further analysis of the spatial distribution of apical cells in Scleractinia planula is necessary.

### Genes Associated With Long Apical Tuft Cilia in Actiniaria

**Fig. 4.**
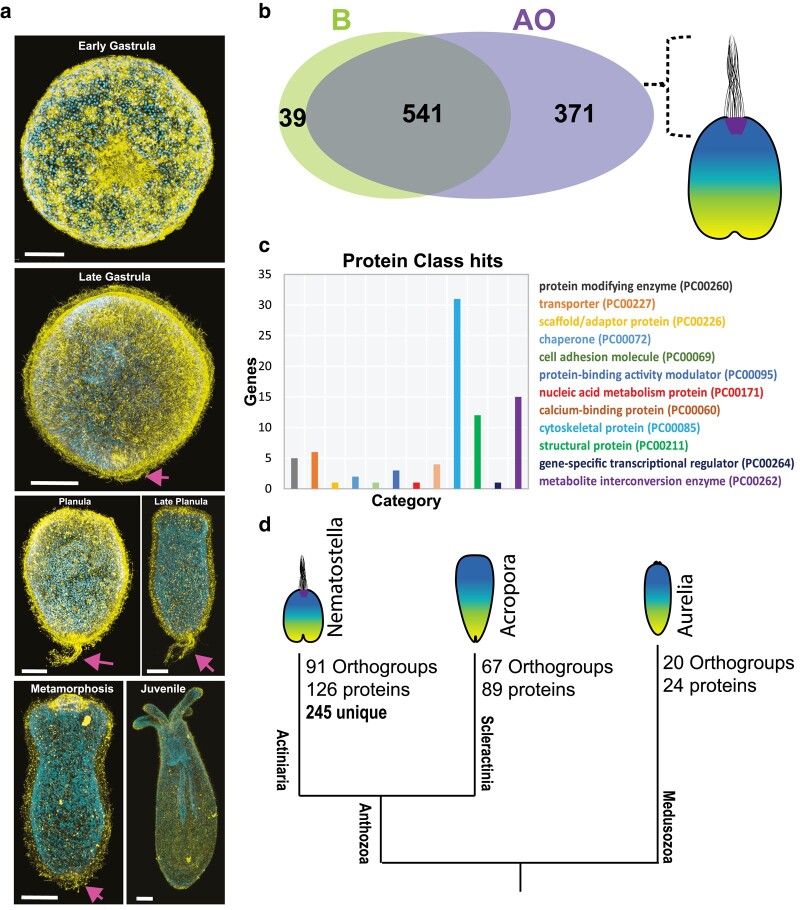
The genes linked with ciliary tuft in cnidaria. a) Different stages of *N. vectensis* development. Immunostaining with the acetylated tubulin antibody and counterstaining with DAPI for nuclei. An arrow pointing to apical tuft. b, c) Tissue-specific transcriptome versus ciliary proteome of *N. vectensis* planula to define cilium-associated genes enriched in apical tuft. b) Venn diagram showing the DEG from the body and apical tissues. c) The apical enriched cilia genes are categorized into different protein classes using gene ontology. d) *N. vectensis* apically enriched cilia orthologs shared with *A. millepora*, *A. tenuis* and *A. aurita*. Out of 371 apically enriched cilia genes, *N. vectensis* uniquely expresses 245 cilia-specific genes in apical domain.

Finally, we investigated if Actiniaria exhibits unique expression of ciliary genes in the apical domain compared to scleractinian and medusozoan planula. The apical tuft cilia are found in a wide range of marine invertebrates such as echinoderms (Yaguchi et al. 2010), molluscs (Dictus and Damen 1997), annelids (Arenas-Mena et al. 2007; Williams and Jekely 2019), and cnidarians (Gilbert et al. 2022). In addition to the ciliary tuft, the ciliated larvae exhibit motile cilia. In general, the motile cilia typically cover the entire body of the larvae, aiding in the organism's movement through ciliary beating (Fig. 4a) The apical tuft does not contribute to larval motility. However, the ciliary tuft in *N. vectensis* displays movement by expanding and contracting into a ciliary bundle. A comparative study of the ciliary proteomes in *N. vectensis* planulae, sea urchins, and choanoflagellates revealed core components of the ciliary intercellular signaling pathways and identified the shared ciliary proteome. The ciliary proteome data were acquired by isolating cilia from whole *N. vectensis* planula, which were subjected to mass spectrometry; this allowed the construction of the ciliary proteome from the whole larvae, including the apical tuft (Sigg et al. 2017).

Here, we integrated the tissue-specific transcriptomes with cilia proteomes from *N. vectensis* planula (Fig. 4b, supplementary file S1, Supplementary Material online). Among the DEGs, 371 ciliary genes were enriched in the apical region, 541 ciliary genes were commonly expressed throughout the body, and 39 were significantly enriched in the body, suggesting that the apical tuft cilia possess a set of proteins distinct from the rest of the body cilia. Among the apical enriched genes, we came across a set of candidates related to cilium organization, cytoskeletal and structural proteins (Fig. 4c), such as dynein heavy chain, axonemal, beta-tubulin, bardet-biedl syndrome, kinesin family member, enkur, tektin ADP ribosylation factor like GTPase, filamin A, tetratricopeptide repeat domain, stabilizer of axonemal microtubules 1, usherin, and intraflagellar transport protein. We also observed genes associated with metabolite interconversion such as WD repeat-containing protein and kinase family members (phosphoenolpyruvate carboxy kinase, nucleoside diphosphate kinase, adenylate kinase) (supplementary file S1, Supplementary Material online).

Finally, we investigated whether Actiniaria exhibits unique expression of ciliary genes in the apical domain compared to scleractinian and medusozoan planulae. Using orthology analysis and spatial transcriptome data we compare the *N. vectensis* apical enriched ciliary genes among *A. millepora*, *A. tenuis* and *A. aurita* apical tissue data (Fig. 4d). Out of 371 ciliary genes enriched in the apical domain of *N. vectensis*, 245 genes were undetected in apical domain of *A. millepora*, *A. tenuis* and *A. aurita* apical tissue (supplementary file S1, Supplementary Material online and supplementary file S6, Supplementary Material online). This suggests that Actiniaria planula innovated a large set of ciliary genes leading to the origin of a unique apical organ with a ciliary tuft. However, it should be noted that some of those 245 ciliary gene orthologs may be present in the genomes of Scleractinia and Medusozoa, but not specifically expressed in the apical domain of planula stage. The crucial factor is the spatial-temporal expression of cilia genes, specifically in the apical domain during the planula stage, which is essential for the development of apical tuft cilia in the Actiniaria.

### A Distinct Molecular Topology of *A. aurita* Apical Domain: Most of the Canonical TFs Associated With Apical Organ GRN are Absent in *A. aurita*

**Fig. 5.**
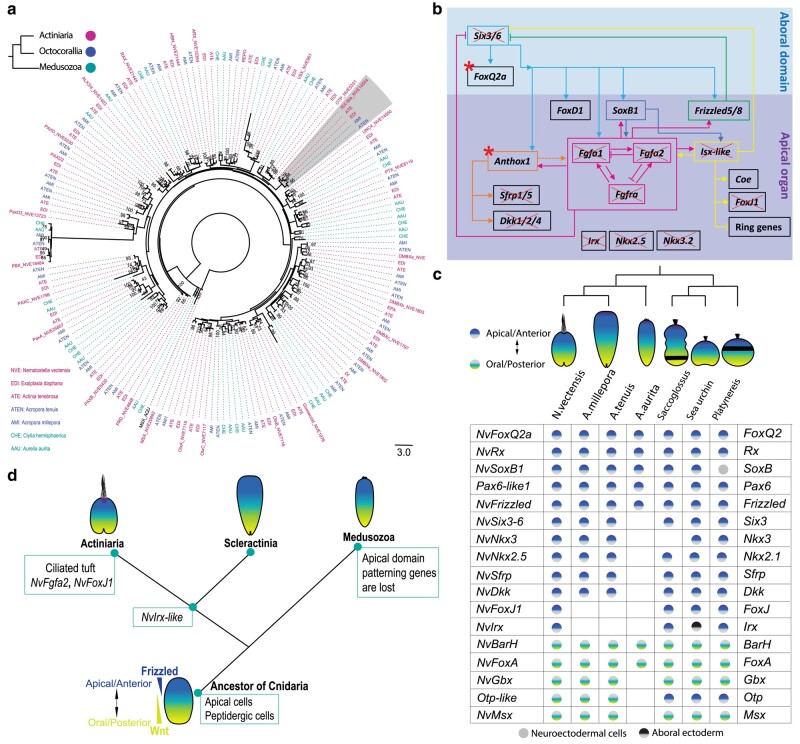
A distinct molecular topology of *a. aurita* apical domain and lack of shared expression of putative neural patterning genes. a) A phylogenetic relationship of cnidarian PRD class proteins. Values on nodes represent Bootstrap values (100 replicates). Bootstrap support values above 50% are indicated above branches. b) Illustrating the apical domain GRN of *N. vectensis* and superimposing the *A. aurita* apical domain genes scenario. The genes that are absent in the apical domain are indicated with a cross. *FoxQ2* and *Anthox1* are expressed but not homologous to *N. vectensis*. c) Shared expression of anteroposterior patterning genes in the larva of cnidarian and bilaterian invertebrate species. Most genes conserved across cnidarian *N. vectensis*, *A. millepora*, *A. tenuis* and bilaterian species lack shared expression in *A. aurita*. d) A possible scenario of apical tuft origin in Cnidaria: the common ancestor of cnidarians possessed apical plates comprised of apical and sensory neurosecretory cells. Apical cells homologs to anthozoans and bilaterians may have lost in the Medusozoa after splitting from Anthozoans. After the split of Actiniaria from other Anthozoans, the Actiniaria innovated genes like *NvFgfa2* and a set of ciliary genes that added a long ciliary tuft to the apical organ.

The Medusozoa is a sister group to the Anthozoa (Fig. 1b). To address the nature of the molecular differences between *N. vectensis* and *A. aurita*, we investigated ortholog data to identify the shared apical domain genes. We found significant expression of *Hox9-14C*, *FoxD1*, *Rx*, *SoxB1*, *Hes2* and *Frizzled* regulatory genes in the *A. aurita* planula apical domain, while *Isx-like*, *Fgfa1*, *Fgfa2*, *Irx, Six3/6, Nkx3*, *Nkx2.5, Tbx4-5*, *Sfrp*, *TauD*, *Dickkopf-like*, *Bmp1-like5, Isl1*, *Erg1* and *Mex3b* genes had no significant differential expression (Fig. 2d). Genes like *Fgfa1* and *Six3/6* showed a lack of minimal read count. Through phylogenetic analysis we confirmed that both *Fgfa2* and *Isx-like* are absent in the genome of Medusozoa (Fig. 3f, Fig. 5a). At least from the orthology analyses, we did not find the orthologs of *Nkx2.5, Not2-like*, *Rfx4-like* and *Bmp1-like5* in the genome of *A. aurita*. In the discussion section, we confer the criticality of some of these genes to highlight their key role in the apical domain and the evolutionary consequences for the apical organ.

## Discussion

### *NvFoxJ1* in the Development of the Apical Tuft Cilia

Among the apical domain genes expressed in the *N. vectensis* apical cells, we observed that *FoxJ1* is expressed explicitly in the *N. vectensis* apical domain, but not in the *A. millepora*, *A. tenuis* and *A. aurita* apical domains. *FoxJ1* is the master regulator of ciliogenesis (Stubbs et al. 2008; Yu et al. 2008; Thomas et al. 2010) and orthologs of *FoxJ1* have been found in vertebrates and invertebrates. TFs, such as FGF (Neugebauer et al. 2009) and Wnt (Caron et al. 2012), act as upstream regulators of *FoxJ1* ciliogenesis (Marlow et al. 2014). A conserved role of *FoxJ1* in motile cilia formation is supported by expression patterns outside chordates. In sea urchin (phylum Echinodermata) larvae, *FoxJ1* expression has been shown in the most apical ectoderm marking the apical tuft (Tu et al. 2006; Yankura et al. 2010; Tisler et al. 2016). Similarly, in annelid *Platynereis dumerilii* and *Owenia fusiformis,* the *FoxJ1* is expressed in the apical plate and the ciliated bands (Seudre et al. 2022). In *N. vectensis*, along with other spot genes, *NvFoxJ1* expression is observed in spot region (supplementary fig. S2, Supplementary Material online) and its expression coincident with the development of the apical tuft (Larroux et al. 2008). Like in bilaterians, the *NvFoxJ1* expression in *N. vectensis* is under the control of FGF signaling (Fig. 2e). KD of *Fgfa1* affects the expression of *NvFoxJ1*, which affects the apical tuft cilia (Rentzsch et al. 2008). Unlike *N. vectensis*, the apical domain of Scleractinia and *A. aurita* planula lack enrichment of *FoxJ1* expression. Furthermore, using the larval single-cell data, we compared *FoxJ1* expression among scleractinian *Stylophora pistillata* (Levy et al. 2021) and actiniarian *N. vectensis* larval cell types (Sebe-Pedros et al. 2018). As indicated in Fig. 3c, supplementary fig. S2, Supplementary Material online, in *N. vectensis*, the *NvFoxJ1* is significantly expressed in the apical organ cell type. In contrast, in *S. pistillata* the *FoxJ1* is enriched in the epidermis but not in the predicted apical cell type (cluster 22 unknown) (Fig. 3d). Jointly, these findings suggest that *FoxJ1* expression in the apical organ of anthozoans likely supported the formation of a long ciliary apical tuft. However, functional studies will have to determine whether *NvFoxJ1* is specifically required for the development of the apical tuft cilia or whether the motile cilia of the body surface also depend on the function of this gene.

### A Distinct Molecular Topology of *a. Aurita* Apical Domain and Lack of Shared Expression of Canonical TFs Associated With Apical Organ GRN

As detailed in the results, unlike *A. aurita* planula apical domain had no significant differential expression of *Isx-like*, *Fgfa1*, *Fgfa2*, *Irx, Six3-6, Nkx3*, *Nkx2.5, Tbx4-5*, *Sfrp*, *TauD*, *Dickkopf-like*, *Bmp1-like5, Isl1*, *Erg1* and *Mex3b* genes (Fig. 2d). Here, we discuss the criticality of some of these genes to highlight their key role in the apical domain and the evolutionary consequences for the apical organ. *NvSix3/6* has a broad role in determining the identity of the aboral domain development. The *Six3/6* antagonistic role in repressing *Wnt* signaling allows the activation of aboral genes, including *FoxQ2*, *Rx*, and *Nkx* (Wei et al. 2009). KD of *NvSix3/6* affects the expression of several apical domain genes, including *NvFGFa1*, *NvFrizzled5/8* and *NvFoxQ2a* (Sinigaglia et al. 2013), which are in turn associated with the expression of downstream genes *NvIrx*, *NvIsx-like*, *NvFgfa2* and *NvFoxJ1* genes, together resulting in a loss of the apical organ and further affecting the larval development (Fig. 2e) (Marlow et al. 2013; Sinigaglia et al. 2013). Likewise, in bilaterians like sea urchins, KD of *Six3* function results in a loss of the apical plate and impairs neural development (Kozmik et al. 2007; Wei et al. 2009). Though *Six3/6* is encoded in *A. aurita* genome (Nakanishi et al. 2015), at the planula stage *Six3/6* is not expressed. Likewise, studies in *Clytia hemisphaerica* identified that *Six3/6* is expressed in both polyp and medusa stages, but not in the planula (Leclère et al. 2019). Strikingly, in *A. aurita*, along with *Six3/6*, many of its downstream genes, including *Irx*, *Isx-like*, *Nkx3*, *Nkx2.5*, *Dkk*, *Fgfa1*, *Fgfa2* and *FoxJ1* genes either lack shared spatiotemporal expression with *N. vectensis* apical domain or absent from the genome of Medusozoa (Fig. 2a, Fig. 5b).

Previously in *N. vectensis*, we identified *NvIsx-like*, a PRD class homeobox gene expressed explicitly in apical tuft cells, as an FGF signaling-dependent TF responsible for forming the apical organ (Gilbert et al. 2022). *Isx-like* KD prevented the formation of the apical tuft cilia and loss of the apical tuft cell identity (Gilbert et al. 2022). From the phylogenetic analysis of the PRD class homeobox gene, we identified that *NvIsx-like* is absent in the medusozoans genome (Fig. 5a, supplementary file S5, Supplementary Material online).

Unlike *Isx-like* homeobox gene, *A. aurita* showed another *Hox* gene expression in the apical domain (Fig. 2a). From the orthology analysis we initially noticed it clustering with NvAnthox1 orthogroup. However, from previous phylogenetic studies in *Clytia hemisphaerica* (Chiori et al. 2009; Steinworth et al. 2022), we understand that it is actually paralogous to NvAnthox1. NvAnthox1 is notable for its apical domain expression in *N. vectensis* (Ryan et al. 2007). A comparative study across anthozoans and medusozoans phylogenetically placed the anthozoan NvAnthox1 clade as a sister to a pair of medusozoan Hox clades, including Hox9-14C (Chiori et al. 2009; Steinworth et al. 2022). Previous studies in hydrozoan species, including *Clytia hemisphaerica* and *Cassiopea xamachana,* have shown *Hox9-14C* expression in apical domain (Chiori et al. 2009; Steinworth et al. 2022). Similarly, the tissue-specific transcriptome of *A. aurita* apical domain showed significant expression of a *Hox* gene homologous to *Hox9-14C*. The consistent pattern of aborally localized expression of *Anthox1/Hox9-14C* in both anthozoans and medusozoans suggests that the cnidarian ancestor utilized *Anthox1/Hox9-14C* signaling in apical domain GRN.

Another well-conserved marker for apical domain territories is the forkhead domain transcription factor *FoxQ2* (supplementary fig. S2, Supplementary Material online), which functions downstream of *Six3/6* in the development of the apical domain of *N. vectensis* (Fig. 2e) (Sinigaglia et al. 2013) and sea urchins (Wei et al. 2009). In *A. aurita*, unlike *Six3/6*, *FoxQ2* shows enrichment in the apical domain. However, as previously identified in *Clytia hemisphaerica*, the *A. aurita* apical enriched *FoxQ2* gene is not an ortholog of *N. vectensis NvFoxQ2a* (Chevalier et al. 2006; Leclère et al. 2019). The GRN around medusozoan *FoxQ2* is yet to be studied. Overall, most of the canonical GRN associated with the *N. vectensis* apical organ either lacks significant expression or is missing from the *A. aurita* genome. Some of these findings also coincide with previous studies in the hydrozoan species *Clytia hemisphaerica* (Chevalier et al. 2006; Chiori et al. 2009; Leclère et al. 2019), which belongs to a separate subgroup of the Medusozoa.

### A Conserved Neuronal Expression Domain map Between Cnidarians Anthozoan and Bilaterians, but not in Medusozoa *a. Aurita*

The frontal region (territory of the apical pole) of cnidarian larvae is set by an apical domain GRN directing the formation of apical organ and subsequent specification of neurons. Developing nervous systems are regionalized by stripes of gene expression along the anteroposterior axis. Along with apical nervous system, the *N. vectensis* planula simultaneously develops neurons at the blastopore/oral end (Kelava et al. 2015; Arendt et al. 2016). Numerous TFs show concentric expression in the oral ectoderm of *N. vectensis* planula larvae, anticipating in development of oral/blastoporal neurons (Marlow et al. 2009, 2013; Mazza et al. 2010; Watanabe et al. 2014) (supplementary fig. S3, Supplementary Material online). Notably, many of these TFs are well-established players in patterning of bilaterian neuroectoderm (Denes et al. 2007; Alaynick et al. 2011; Hejnol and Lowe 2015), expressed in a similar sequence of domains in annelids (Denes et al. 2007), cephalochordates (Mazet and Shimeld 2002; Gattoni et al. 2023) and hemichordates (Lowe et al. 2003). The expression of nervous system genes can be segregated into three broad groups to facilitate the comparison between cnidarians and bilaterians: anterior, midlevel, and posterior genes. As shown in the supplementary fig. S3, Supplementary Material online, at the anterior domain, 13 genes were identified in *N. vectensis*, namely *NvSix3/6*, *NvFoxQ2*, *NvRx*, *NvFoxD1*, *NvOtx*, *NvFrizzled5/8*, *NvNkx2.5*, *NvNkx3.2*, *NvIrx*, *NvDkk*, *NvSfrp1/5*, *NvSoxB1* and *NvFoxJ1*. Most of these genes are known to express within the forebrain or anterior territory of hemichordates (Holland et al. 2013) and they all have prominent expression domains in the prosome ectoderm of *S. kowalevskii*, the hemichordate's most anterior body part (supplementary fig. S3, Supplementary Material online) (Lowe et al. 2003; Fritzenwanker et al. 2014; Lowe 2021); these ortholog cognates express entirely within the apical domain in *N. vectensis*. Midlevel genes are those expressed in the mesosome and anterior metasome (with some domains extending anteriorly into the prosome), that is, more posteriorly than those genes of the anterior group. In case of hemichordates, they expressed at least in the midbrain, having posterior boundaries in the midbrain or anterior hindbrain (supplementary fig. S3, Supplementary Material online). In *N. vectensis*, at the midlevel ten genes were identified, namely *NvPax6*, *NvIrx*, *NvDbx*, *Nvlim1/5*, *NvMsx*, *NvDlx*, *NvDll*, *NvWnt2* and *NvWnt8*. Some of these genes differ in their anterior and posterior extent (supplementary fig. S3, Supplementary Material online). Posterior genes are those expressed entirely within the hindbrain and spinal cord regions of the chordate nervous system. In hemichordates, they share orthologous expression in the posterior metasome (Lowe et al. 2003; Fritzenwanker et al. 2014; Darras et al. 2018). At the posterior domain, 12 genes were compared, namely *NvGbx*, *NvBrah*, *NvMsx*, *NvOtp*, *NvOtx*, *NvFoxA*, *NvAnthox6*, *NvWntA*, *NvWnt1*, *NvWnt3*, *NvWnt4* and *NvWnt16* as shown in the supplementary fig. S3, Supplementary Material online. All these genes are expressed in the oral region of *N. vectensis*. Despite the significant phylogenetic distance, the relative order in which the axial TFs are expressed in *N. vectensis* is similar to the order of their expression along the axis of the bilaterian nervous system development (supplementary fig. S3, Supplementary Material online).

Taking these nested concentric domains of *N. vectensis* genes, we asked whether these orthologous genes are expressed in a similar pattern along the anterior–posterior axis in other cnidarian groups (Schwaiger et al. 2022; Formery et al. 2023). Using spatial transcriptome, we can only distinguish the expression of a gene enriched in the apical domain or the rest of the body. Therefore, to facilitate the comparison, we combined the midlevel and posterior genes in *A. aurita*, *A. millepora* and *A. tenuis*. As shown in the Fig. 5c, we indicated the list of shared neuronal genes across the anterior and midlevel/posterior domains of planula. Comparing the orthologs between Actiniaria and Scleractinia larvae show majority of genes with in similar spatial expression pattern (Fig. 5c). In contrast, the *A. aurita* larvae lack shared expression of *Irx*, *Isx-like*, *Six3/6*, *Nkx3*, *Nkx2.5* and *Dkk* genes, while *FoxQ2*, *Rx*, *Frizzled5/8*, *Sfrp1/5*, *SoxB1*, *Brah*, *FoxA*, *Wnt1*, *Wnt3*, *Wnt4* and *Wnt16* have shown the similar expression pattern with *N. vectensis* (Fig. 2d, Fig. 5c). Thus, in bilaterians, as in cnidarians, *Gbx*, *Msx*, *BarH*, *Pax*, *Dbx*, *FoxA*, *FoxB* and *Lim* members are expressed in the lateral region. In the case of *A. aurita*, the *Msx* and *Dbx* are absent in posterior domain, whereas *Gbx* and *Pax3-7/PaxD* are absent in the genome (Gold et al. 2019; Khalturin et al. 2019a). Along both anterior and posterior domains, the *A. aurita* showed variations in GRN; several crucial apical organ and neuronal genes along the anteroposterior axis are lacking conserved expression with *N. vectensis*. Hence, most of these genes are evolutionarily conserved between anthozoans and bilaterians (supplementary fig. S3, Supplementary Material online) and are associated with anteroposterior neurogenesis and axial formation suggesting that *A. aurita* has undergone extensive GRN changes after the anthozoan split.

## Conclusion

The inconsistency of the apical organ with ciliary tuft among cnidarians provides a window to understand the evolution of apical organ and the GRN that operated in the common ancestor of eumetazoans (cnidarian and bilaterian ancestor). Comparative gene expression studies between the cnidarian *N. vectensis* and bilaterian ciliated larvae revealed a strong resemblance in the molecular topography around the apical pole (Matus et al. 2006; Sinigaglia et al. 2013; Marlow et al. 2014; Arendt et al. 2016), suggesting that the apical organ may be an evolutionarily conserved larval structure and might have appeared within the ancestor of Eumetazoa. While the apical domain GRN is extensively studied in *N. vectensis* and compared with bilaterians, the evolutionary relationship of the apical domain within cnidarian groups remains unknown. Here, we utilized the well-defined developmental TFs found in the *N. vectensis* GRN as a framework to reveal how these conserved regulatory interactions have shifted in the apical domain of different cnidarian groups. Despite the morphological diversity between Actiniaria and Scleractinia planula primarily lacking apical tuft, the *A. millepora* and *A. tenuis* larvae share an extensive gene profile with *N. vectensis* in the apical domain. The shared gene composition with *N. vectensis*, thereby shared apical signaling system, reflects homology. Even though shared TFs unite Actiniaria and Scleractinia planula, the architecture of apical domain and ciliary transcriptome are considerably different.

Next, we showed that genes involved in patterning the apical domain of anthozoan larvae are mainly absent in the stem leading to Medusozoa planula, suggesting that the scyphozoan lacks apical organ homologs to anthozoans; this implies a dramatic reorganization of GRN in Medusozoa apical domain. Along the anteroposterior axis, the *A. aurita* showed drastic changes in GRN specifying oral-aboral identity; several crucial apical organ and neuronal genes along the anteroposterior axis are lacking shared expression with *N. vectensis*. Strikingly, most of these genes are evolutionarily conserved between anthozoans and bilaterians, associated with anteroposterior neurogenesis and axial formation. That suggests that *A. aurita* has undergone extended changes in GRN after the split from anthozoans. It might be early to determine this based on current transcriptome data from tissue-specific transcriptome alone. On the other hand, some of our results accord with previous findings in a hydrozoan species, *Clytia hemisphaerica*, suggesting that the medusozoan larvae are indeed much simpler than the anthozoan. Previous studies in comparison of molecular data proposed that anthozoan polyps, medusozoan polyps and a jellyfish stage are equally different from one another and suggested that the only truly conserved stage among the Anthozoa and Medusozoa might be the planula larva, which becomes the best candidate for the cnidarian ancestral body plan (Khalturin et al. 2019a). However, our data demonstrate that the planulae of anthozoans and medusozoans are also highly different.

With this study, we provide crucial insights into the molecular signature of the larval sensory structure across the Cnidaria and its evolutionary history. Based on the earlier studies in scleractinian and medusozoan planulae, it is clear that neuropeptide-expressing cells are present in all groups of cnidarians. We show that the scleractinian planulae share most apical domain GRN with *N. vectensis*, suggesting that scleractinian planulae have apical cells homologous to *N. vectensis*. On other hand, medusozoans potentially lack an apical organ that is homologous to anthozoans and bilaterians. It is plausible that the common ancestor of cnidarians possessed an apical plate comprised of apical and sensory neurosecretory cells and that after the split of Actiniaria from other anthozoans, the Actiniaria planula innovated cilia-associated genes leading to the origin of apical organ with a ciliary tuft (Fig. 5d).

## Methods

### Animal Collection and Culturing

*Nematostella vectensis*: Polyps were grown in 16 ‰ artificial seawater at 18 °C in the dark and fed with freshly hatched *Artemia* nauplii. The induction of spawning was performed as previously described (Genikhovich and Technau 2009b). After fertilization, the gelatinous substance around the eggs was removed using 4% L-Cysteine (Sigma-Aldrich, USA) (Genikhovich and Technau 2009b). *Aurelia aurita*: Ephyrae were collected using a plankton net in the vicinity of Plymouth Sound, UK and cultured in seawater at 18 °C in a 12:12 light and dark cycle. The jellyfish were fed twice a day with freshly hatched *Artemia nauplii*. Fertilized embryos were collected from the brood sacs and reared to the planula stage. *Acropora millepora* and *Acropora tenuis*: The corals were cultured in ex-situ (Craggs et al. 2017, 2020). During the annual spawning of 2020, the embryos were collected after fertilization. The larvae are maintained in artificial seawater at 27 °C and collected for experimentation at the planula stage.

### Microdissection of Planula Larvae

To separate the apical region from the rest of the larval body, we performed microdissection on *N. vectensis*, *A. aurita, A. millepora* and *A. tenuis* larvae as described in our previous study in *N. vectensis* (Gilbert et al. 2022). Apical tissue containing the apical organ was isolated using 34-gauge needles under a stereomicroscope with 10× magnification. Motile larvae were placed into a fresh plastic Petri dish filled with *N. vectensis* medium or filtered seawater. The larvae tend to adhere briefly to the bottom of a new plastic Petri dish, allowing enough time to separate the apical tissue by cutting. Each sample was pooled from a minimum of 100 individual larvae. For each planula prior to dissection, the frontal region is identified by means of the direction of larvae swimming. The samples were carefully collected using glass Pasteur pipettes, the excess medium was removed, and the samples were snap-frozen in liquid nitrogen and stored at −80 °C until further processing.

### RNA Sequencing and Differential Gene Expression

For RNA isolation, due to the sheer size, samples collected from multiple batches were combined to acquire an adequate amount of RNA for sequencing. Total RNA was isolated using the TRI Reagent® according to the manufacturer's protocol. RNA quality was assessed using Agilent RNA 6,000 Nano Kit on Agilent 2,100 Bioanalyzer (Agilent, USA), and samples with RNA integrity number ≥ 8.0 were used for sequencing. The CORALL RNA-Seq Library Prep Kit (Lexogen GmbH) was used for library preparation. Before sequencing, the libraries were pre-assessed by Agilent High Sensitivity DNA Kit (Agilent, USA) and quantified using Qubit™ 1× dsDNA HS Assay Kit (Invitrogen™). The sequencing was outsourced (GENEWIZ Illumina NovaSeq™ 2 × 150 bp sequencing), generating 15 million paired-end reads per replicate. Raw files and processed data were deposited at NCBI GEO submission GSE242174. After de-multiplexing and filtering high-quality sequencing reads, the adapter contamination was removed using *fastp* an ultra-fast all-in-one FASTQ preprocessor (Chen et al. 2018). Furthermore, the quality of the reads was verified using FastQC (Simon 2010). Processed reads from each sample were mapped to the respective genome and gene models [indexed bowtie2 (Langmead and Salzberg 2012)] by using STAR (Spliced Transcripts Alignment to a Reference) (Dobin et al. 2013). *N. vectensis* gene model (https://figshare.com/articles/Nematostella_vectensis_transcriptome_and_gene_models_v2_0/807696). *A. aurita* (Khalturin et al. 2019b), Coral *A. millepora* (Ying et al. 2019) and *A. tenuis* (Shinzato et al. 2021). The number of reads mapping to the respective gene model were extracted from STAR output using the featureCounts tool (Liao et al. 2014). Differential expression analyses were performed using DESeq2 (Galaxy Version 2.11.40.7 + galaxy2) (Love et al. 2014).

### Identification of Orthologs

We performed a BLASTP (Altschul et al. 1997) search with the default curated gathering threshold for functional annotation to predict the protein homologs against the UniProt database (UniProt 2015). Additionally, we used gene functional annotation data from the published studies of respective species: *N. vectensis* single-cell transcriptome study (Sebe-Pedros et al. 2018; Steger et al. 2022) and *A. aurita* genome study (Gold et al. 2019; Khalturin et al. 2019a) (supplementary file S1, Supplementary Material online). To identify genome-wide orthologous clusters across selected cnidarian species, we used OrthoVenn3 (https://orthovenn3.bioinfotoolkits.net/home) (Wang et al. 2015; Sun et al. 2023), a genome-wide comparison and visualization tool. OrthoVenn3 is an integrated web-based platform for exploring and visualizing orthologous data across genomes. OrthoVenn3 employed the OrthoMCL algorithm for comparative analysis of orthologous clusters (Wang et al. 2015; Sun et al. 2023). Protein sequences of the reference genomes were organized into orthologous gene groups based on sequence similarity (Wang et al. 2015). The whole-genome protein sequences of all species were checked for sequences containing characters other than amino acids in the fasta file, and sequences smaller than <20 amino acids were removed. We retrieved the protein sequences of all gene coding sequences without alternative splice variants. The OrthoMCL performs an all-against-all BLASTP alignment, identifies putative orthology and in-paralogy relationships with the Inparanoid algorithm (Remm et al. 2001) and generates disjoint clusters of closely related proteins with the Markov Clustering Algorithm (MCL) (Enright et al. 2002). We primarily used the OrthoMCL output as a reference to narrow down our search to orthologous groups and to identify putative orthology matches. Next, we validated the identified putative orthology match by reciprocal blast search with the respective genome. Furthermore, to deduce the putative function of each ortholog, the first protein sequence in each cluster are subjected to BLASTP analysis against the non-redundant protein database in UniProt (UniProt 2015).

### Phylogeny

For FGF and PRD class homeobox phylogenetic analysis, the following species were selected from Actiniaria: *N. vectensis*, *Exaptasia pallida*, Scleractinia: *A. millepora* and *A. tenuis*, and Medusozoa: *Clytia hemispherica* and *A. aurita.* The protein sequences considered for the FGF and PRD class hox homologs search was provided in the supplementary file S5, Supplementary Material online. We carried out BLASTP searches using the published transcriptome data of each selected cnidarian species. In case we found no matches, we carried out TBLASTN searches with the same query sequences. The sequences were obtained from orthology analysis or blast search. The protein sequences were aligned using MUSCLE (Edgar 2004) algorithm in the SeaView program (Gouy et al. 2021). The maximum-likelihood (ML) phylogenetic trees were constructed using PhyML 3.0 online (www.atgc-montpellier.fr/phyml/) (Guindon et al. 2010). The model was automatically selected by the Smart Model Selection with (SH-aLRT) (Lefort et al. 2017). Statistical tree robustness was assessed in PhyML via 100 bootstrap replicates.

### In Situ Hybridization (ISH)

ISH was performed according to published protocols (Genikhovich and Technau 2009a; Wolenski et al. 2013). In brief, fixed animals were transferred into sieves and rehydrated in 1 mL 60% methanol/40% PBST and then washed in 30% methanol/70% PBST. Samples were digested in proteinase K (80 µg/mL) for 5 min then blocked in glycine (4 mg/mL). Larvae were then transferred into 4% formaldehyde at RT for 1 h. Hybridization was carried out with DIG-labeled probes for 48 h at 60 °C. After incubation, samples were washed through serial dilutions of 25%, 50%, 75%, 100% 2 × SSCT at hybridization temperature. The color development was carried out in a 1:50 dilution of NBT/BCIP at RT. Stained animals were visualized with a Leica DM1000 microscope equipped with a MC190 HD Microscope Camera (Leica, Germany). For each gene at least 30 specimens were tested.

### Whole-mount Immunofluorescence and SEM

After fixation, the samples were washed 5 times with PBST [1× PBS, 0.05% (vol/vol) Tween-20] for 10 min. The samples were blocked in 5% BSA in PBST for 1 h at RT. Primary antibody (1:500 dilution, mouse Anti-α-Tubulin Cat # T9026, Sigma-Aldrich) incubation was performed in a blocking solution (1% BSA in PBST) for 24–36 h at 4 °C. The samples were washed with PBST for 5 × 5 min, after which samples were incubated with secondary antibodies (1:250 dilution; Goat anti-Mouse IgG Alexa Fluor 594 Cat # A-11032, ThermoFisher) diluted in blocking solution for overnight at 4 °C. Then, the samples were washed with PBST for 5 × 10 min. Imaging was performed on Leica TCS SP8 DLS and Leica DMi8 confocal microscopes. Sample preparation for scanning electron microscopy was performed as in (Kraus et al. 2016); SEM imaging was performed using the JEOL IT 300 scanning electron microscope.

## Supplementary Material

msad285_Supplementary_Data

## Data Availability

The data underlying this article are available in the article and in its online Supplementary material. The bulk RNA sequencing data has been submitted to GEO and can be accessed using the accession number GSE242174.
