# Supplementary material for: Gene Regulatory Network that Shaped the Evolution of Larval Apical Organ in Cnidaria
Source: Mol Biol Evol. 2023 Dec 28;41(1):msad285. doi: 10.1093/molbev/msad285 (PMC10781443; doi:10.1093/molbev/msad285)

Gene regulatory network that shaped the evolution of larval apical organ in Cnidaria

Authors:

Eleanor Gilbert[1, 2], Jamie Craggs[3], Vengamanaidu Modepalli[1*]

Supplementary data:

Supplementary Figure S1: Illustrating *N. vectensis* oral/posterior domain GRN. In the posterior region, a set of genes are enriched in the central domain (yellow), and others adjust to oral (green) (Rottinger, et al. 2012; Layden, et al. 2016; Lebedeva, et al. 2021).

Supplementary Figure S2: ISH of apical organ-enriched TFs

Supplementary Figure S3: Expression of nervous system axial patterning genes across cnidarians and bilaterians (Lowe, et al. 2003; Steinmetz, et al. 2010; Yankura, et al. 2010; Sinigaglia, et al. 2013; Fritzenwanker, et al. 2014; Marlow, et al. 2014; Arendt, et al. 2016; Achim, et al. 2018; Darras, et al. 2018; Khadka, et al. 2018; Feuda and Peter 2022; Faltine-Gonzalez, et al. 2023). Schematic representation of the anteroposterior expression domains of genes in cnidarian *N. vectensis* and a similar set of genes known to deploy in anteroposterior neuronal patterning in bilaterian species.

Supplementary File 1: Data relevant to figure 1, 2, 3, 4

Supplementary File 2: Orthanalysis Analysis (Data related to Figure. 1E-G)

Supplementary File 3: Orthanalysis Analysis (Data related to Figure. 2A-D)

Supplementary File 4: *S. pistillata* vs *N. vectensis* Orthanalysis Analysis (Data related to Figure. 3A and B)

Supplementary File 5: FGF and PRD class homeobox phylogenetic analysis (Data related to Figure. 3G and 5A)

Supplementary File 6: *N. vectensis* apical domain cilia orthanalysis analysis (Data related to Figure. 4)

Reference:

Achim K, Eling N, Vergara HM, Bertucci PY, Musser J, Vopalensky P, Brunet T, Collier P, Benes V, Marioni JC, et al. 2018. Whole-Body Single-Cell Sequencing Reveals Transcriptional Domains in the Annelid Larval Body. Mol Biol Evol 35:1047-1062.

Arendt D, Tosches MA, Marlow H. 2016. From nerve net to nerve ring, nerve cord and brain--evolution of the nervous system. Nat Rev Neurosci 17:61-72.

Darras S, Fritzenwanker JH, Uhlinger KR, Farrelly E, Pani AM, Hurley IA, Norris RP, Osovitz M, Terasaki M, Wu M, et al. 2018. Anteroposterior axis patterning by early canonical Wnt signaling during hemichordate development. PLOS Biology 16:e2003698.

Faltine-Gonzalez D, Havrilak J, Layden MJ. 2023. The brain regulatory program predates central nervous system evolution. Scientific Reports 13:8626.

Feuda R, Peter IS. 2022. Homologous gene regulatory networks control development of apical organs and brains in Bilateria. Science Advances 8:eabo2416.

Fritzenwanker JH, Gerhart J, Freeman RM, Lowe CJ. 2014. The Fox/Forkhead transcription factor family of the hemichordate Saccoglossus kowalevskii. EvoDevo 5:17.

Khadka A, Martínez-Bartolomé M, Burr SD, Range RC. 2018. A novel gene's role in an ancient mechanism: secreted Frizzled-related protein 1 is a critical component in the anterior–posterior Wnt signaling network that governs the establishment of the anterior neuroectoderm in sea urchin embryos. EvoDevo 9:1.

Layden MJ, Rentzsch F, Röttinger E. 2016. The rise of the starlet sea anemone Nematostella vectensis as a model system to investigate development and regeneration. WIREs Developmental Biology 5:408-428.

Lebedeva T, Aman AJ, Graf T, Niedermoser I, Zimmermann B, Kraus Y, Schatka M, Demilly A, Technau U, Genikhovich G. 2021. Cnidarian-bilaterian comparison reveals the ancestral regulatory logic of the β-catenin dependent axial patterning. Nature Communications 12:4032.

Lowe CJ, Wu M, Salic A, Evans L, Lander E, Stange-Thomann N, Gruber CE, Gerhart J, Kirschner M. 2003. Anteroposterior patterning in hemichordates and the origins of the chordate nervous system. Cell 113:853-865.

Marlow H, Tosches MA, Tomer R, Steinmetz PR, Lauri A, Larsson T, Arendt D. 2014. Larval body patterning and apical organs are conserved in animal evolution. BMC Biol 12:7.

Rottinger E, Dahlin P, Martindale MQ. 2012. A framework for the establishment of a cnidarian gene regulatory network for "endomesoderm" specification: the inputs of ss-catenin/TCF signaling. PLoS Genet 8:e1003164.

Sinigaglia C, Busengdal H, Leclere L, Technau U, Rentzsch F. 2013. The bilaterian head patterning gene six3/6 controls aboral domain development in a cnidarian. PLoS Biol 11:e1001488.

Steinmetz PR, Urbach R, Posnien N, Eriksson J, Kostyuchenko RP, Brena C, Guy K, Akam M, Bucher G, Arendt D. 2010. Six3 demarcates the anterior-most developing brain region in bilaterian animals. EvoDevo 1:14.

Yankura KA, Martik ML, Jennings CK, Hinman VF. 2010. Uncoupling of complex regulatory patterning during evolution of larval development in echinoderms. BMC Biol 8:143.

Supplementary Figures

Supplementary Figure 1

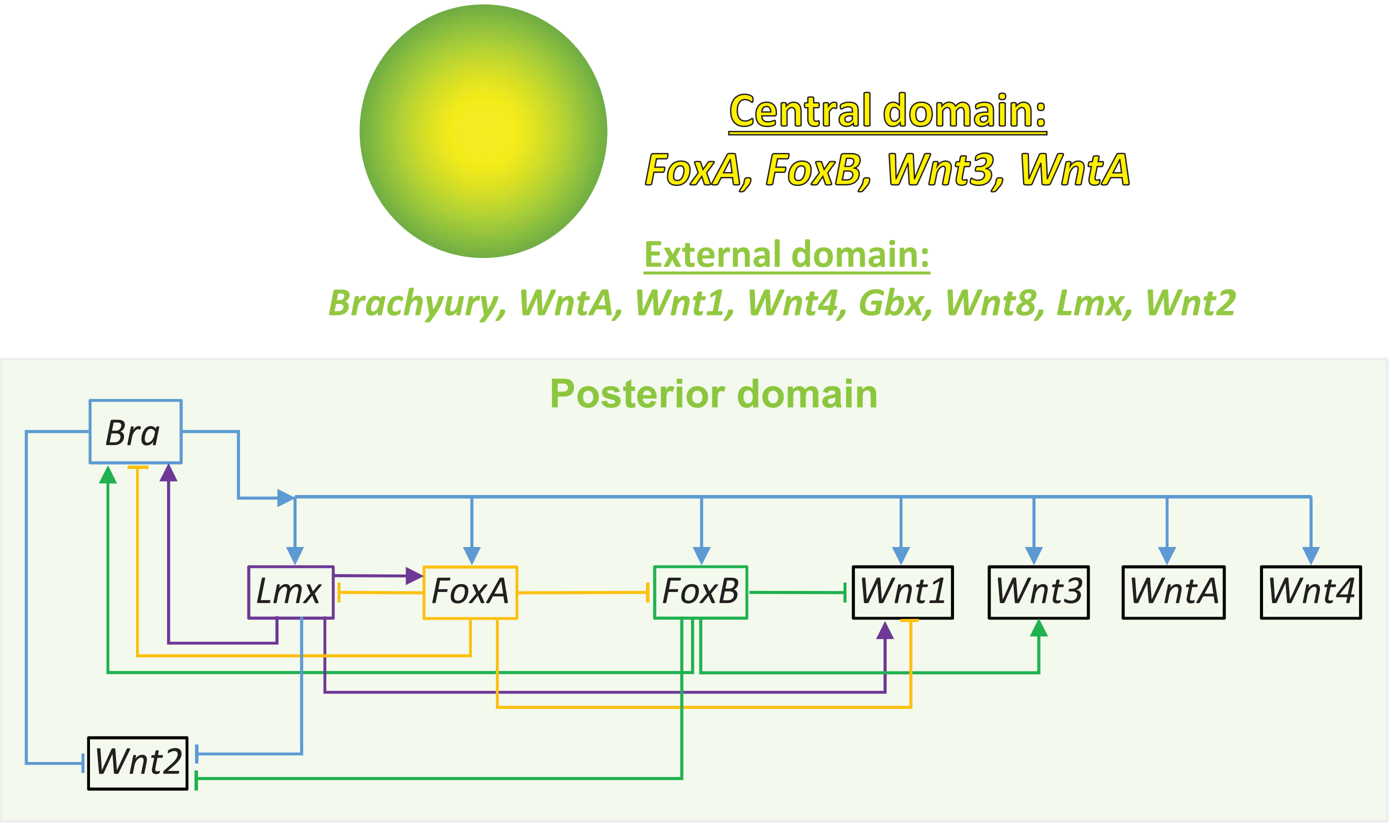

Supplementary figure 2

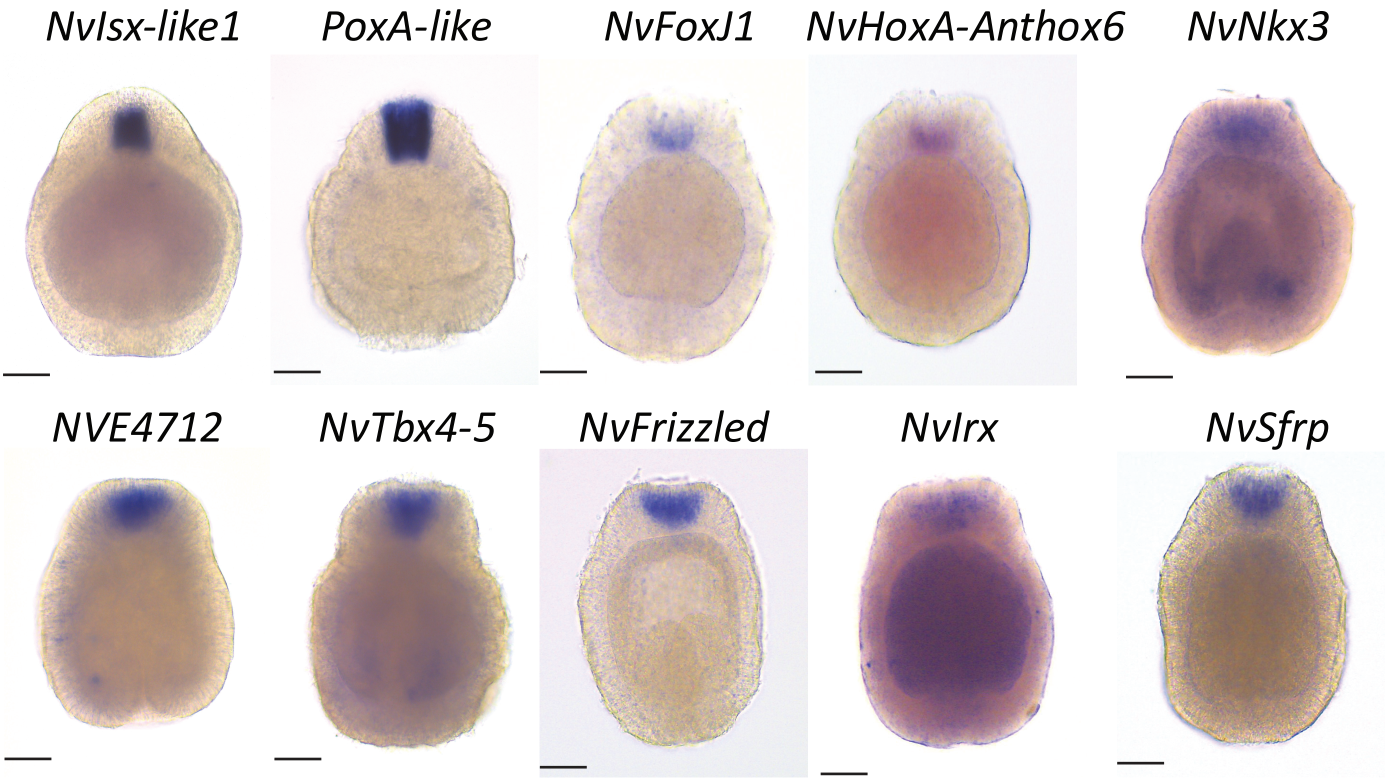

Supplementary Figure 3

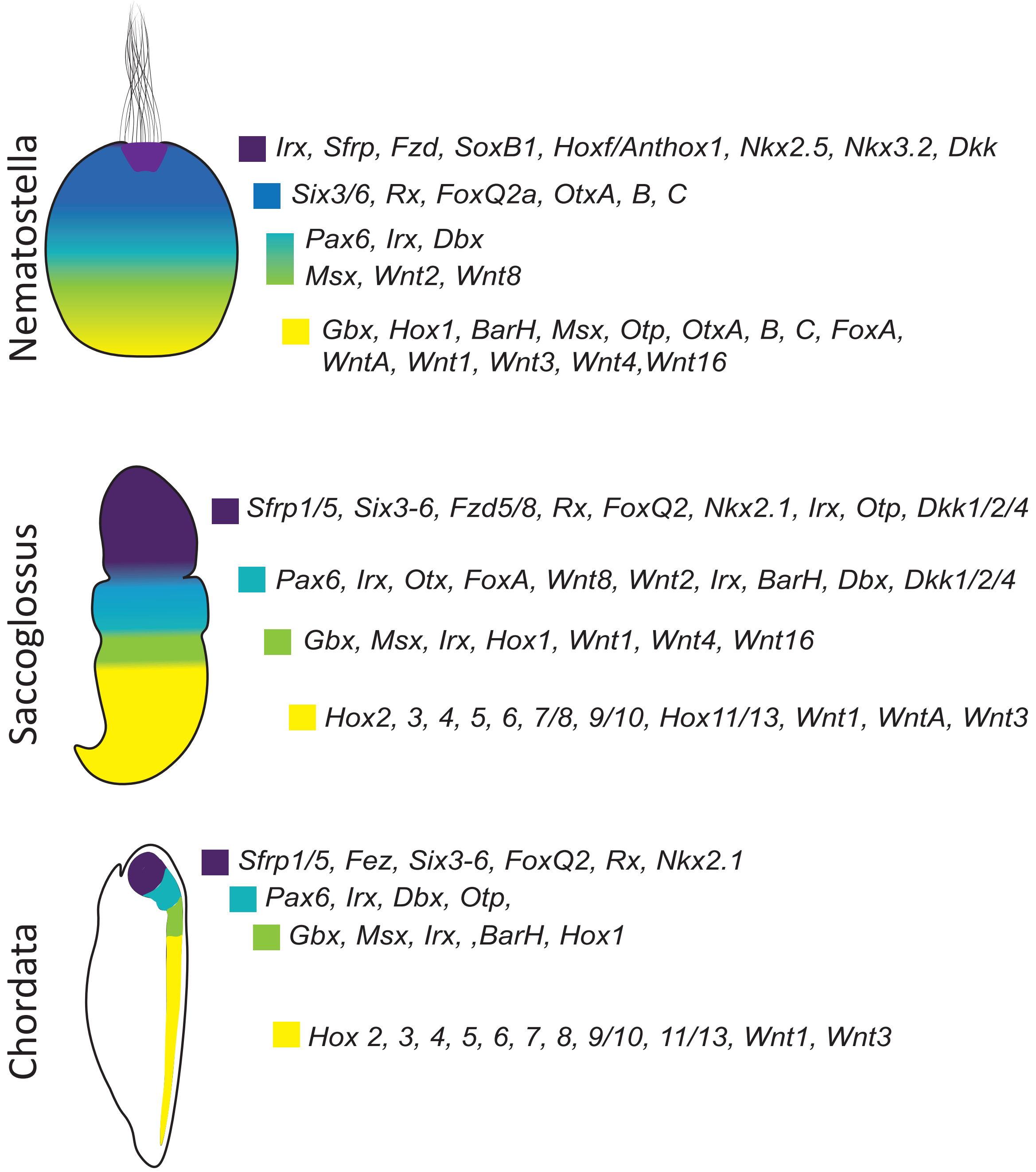

Supplement: msad285_Supplementary_Data [file msad285_supplementary_data.zip › Supplementary data.pdf]
